# DNA barcoding of *Schizothorax* species from the Neelum and Jhelum Rivers of Azad Jammu and Kashmir

**DOI:** 10.1080/23802359.2016.1258337

**Published:** 2016-12-09

**Authors:** Tasleem Akhtar, Ghazanfar Ali

**Affiliations:** Department of Biotechnology, University of Azad Jammu and Kashmir, Muzaffarabad, Pakistan

**Keywords:** Mitochondrial genome, Schizothoracinae, *CO1* gene, river Neelum and Jhelum

## Abstract

The mitochondrial *Cytochrome Oxidase 1* gene is used as a standardized, authenticated, and reliable genetic marker for a global species-level bio-identification system. The present study was conducted to determine whether barcoding can help accurate species identification in fishes. The overall base composition of *Schizothorax* species was 29.6% of T, 25.5% of C, 26.5% of A, and 18.4% of G, A + T content 56.1% and G + C content 43.9%. The *T*s/*T*v bias (*R*) was 2.51. Complete *COI* gene was amplified using PCR and sequenced from 17 samples collected from river Neelum and Jhelum, and identification of species were done by following Mirza (1991), Jhingran (1991) classification and also through BOLD (99.3 to 99.9%) and NCBI (99.6 to 99.9%) reference sequences of those species. Multiple alignments of *CO1* mtDNA gene resulted in a range of 1535–1551 base pairs. Out of 1535 consensus sites, 1490 were constant, 61 characters were variable, in which 54 were parsimony informative, and 7 variables were parsimony uninformative. This is the very first study reported from a reservoir of cold water bodies of Azad Kashmir which have a great potential for conservation of cold water fish species. We emphasized that, DNA barcoding is an accurate, reliable and has the great potential for identification of freshwater fish species.

Accurate identification of fishes is very important in many areas and would improve the fish conservation and ecosystem research and contribute for long-term fish management and its sustainability. For this purpose, a large variety of DNA and protein-based methods has been used. A short fragment of *CO1* gene (655 bp) has been used for identification of species from 30 years, but in different laboratories, different DNA sequences have been also used for species identification (Hebert et al. 2003). The family Cyprinidae containing the genus *Schizothorax*, are locally known as snow trout, containing 20 genera and more than 150 species throughout the world (Mirza 1991). The genus *Schizothorax* contains the remarkably similar morphology, and difficult to distinguish based on the external morphological characters. For the current study, *Schizothorax* samples were collected from the river Neelum and Jhelum Azad Kashmir, Pakistan (34°23′03.0″N) and (73°27′53.8″E). Total DNA was isolated by standard phenol–chloroform extraction by Sambrook et al. (1989). Sequencing primers of complete *CO1* gene of *Schizothorax* species were designed by using program Primer-3. After sequencing and alignment, these sequences were deposited in Genbank for accession numbers (Table 1). The nucleotide composition, nucleotide and haplotype diversity and neutrality test were examined by MEGA6 (Tamura et al. 2013) and DnaSP 5.0 program.

**Table 1.** List of samples used in this study, including species name, code, sample locality and Genbank Accession numbers.

No	Species name	Code	Sex	Genbank accession no.	Collection locality
1	*S. plagiostomus*	SP-AM	Male	KU317684	River Jhelum, Air Port
2	*S. plagiostomus*	SP-AX	Female	KU317689	River Jhelum, Kohala
3	*S. plagiostomus*	SP-BH	Male	KU317694	River Jhelum, Domel
4	*S. plagiostomus*	SP-AT	Male	KU317687	River Jhelum, Domel
5	*S. plagiostomus*	SP-AV	Male	KU317688	River Jhelum, Chatter Kalass
6	*S. plagiostomus*	SP-AZ	Female	KU317690	River Jhelum, Chatter Kalass
7	*S. plagiostomus*	SP-BG	Male	KU317693	River Jhelum, Chatter
8	*S. esocinus*	SE-AL	Male	KU317698	River Jhelum, Ambor
9	*S. esocinus*	SE-BA	Female	KU317699	River Jhelum, Ambor
10	*S. esocinus*	SE-BB	Female	KU317700	River Jhelum, Ambor
11	*S. esocinus*	SE-BE	Male	KU317701	River Jhelum, Ambor
12	*S. esocinus*	SE-H	Male	KU317702	River Neelum, Chella Bandi
13	*S. niger*	SN-AS	Female	KU317703	River Jhelum, Domel
14	*S. niger*	SN-AW	Female	KU317704	River Jhelum, Domel
15	*S. progastus*	SPR-AU	Female	KU317705	River Jhelum, Garhi Dupatta
16	*S. progastus*	SPR-AY	Male	KU317706	River Jhelum, Garhi Dupatta
17	*S. progastus*	SPR-BC	Female	KU317707	River Jhelum, Garhi Dupatta

A total of 17 *COI* barcodes were obtained from four species of Schizothorax. The absence of stop codons and well defined peaks indicated that co-amplification of nuclear pseudo-genes did not occur (Zhang & Hewitt 1996). The total 17 sequences of the same species were downloaded from Genbank (NCBI). Multiple alignments of *CO1* gene resulted in a range of 1535–1551 base pairs. Out of 1535 consensus sites, 1490 were constant, 61 characters were variable, in which 54 were parsimony informative, and 7 variables were singleton. The nucleotides of *CO1* gene sequenced were globally G-deficient (18.4%), whereas (A, 26.5%; C, 25.5%; T, 29.6%). Such type of nucleotide composition pattern has been widely stated in many other fish species with the smaller variations (Khan et al. 2015). The A + T content 56.1% and G + C content 43.9% showing an obvious anti-G bias as appear commonly in teleost fishes (Zhu et al. 2012). The average percentage divergence (K2P) distance of individual’s species of *S. plagiostomus* is 0.003% and 0.002% for *S. esocinus* and 0.001% for *S. niger* and *S. progastus.* There is high inter-specific sequence divergence for studied *Schizothorax* species i.e. 0.009% as compared to intra-specific sequence divergence. The possibility of inter-specific sequence divergence is due to hybridization and ancestral polymorphisms (Hajibabaei et al. 2006). The lack of difference in the mitochondrial sequence data of some *Schizothorax* species may be explained in terms of introgressive hybridization, incomplete lineage sorting, rapid radiation in lineages, and multiple hits (homoplasy) (Qi et al. 2007).

The neighbour-joining (Figure 1) analysis of the *COI* barcode region placed *S. plagiostomus* and *S. esocinus* as sisters to *S. progastus* while, *S. niger* form a separate cluster. As the *S. niger* inhabiting cold streams and rivers is distributed in the inland waters of occupied Kashmir (Kullander et al. 1999), but in present study *S. niger* was first time collected from river Jhelum near Muzaffarabad city. This fish is restricted to upper part of river Jhelum due to damming of waters. However, fortunately due to floods, the eggs and larvae of *S. niger* migrated from upper (Eastern) side of river Jhelum (India) toward its lower side (Pakistan).

**Figure 1.**
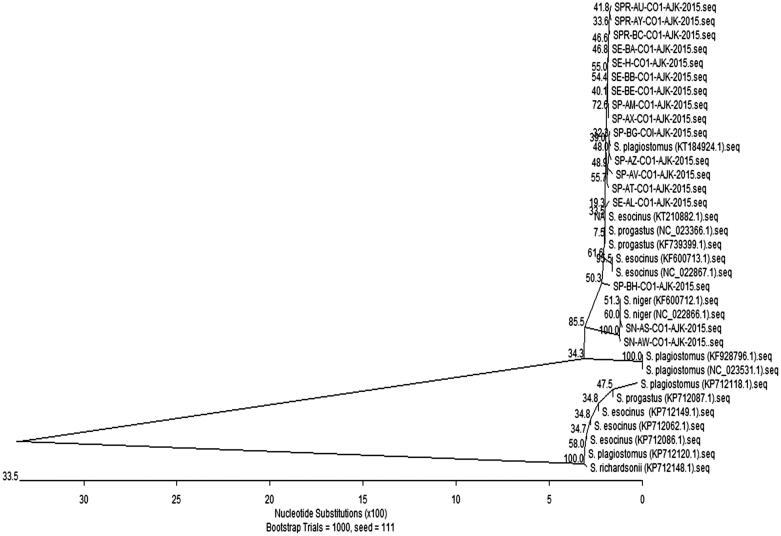
The phylogenetic analysis of *Schizothorax* species of present study and international sequences (with accession number) by neighbour-joining method using MegAlign program (DNASTAR).

*CO1* gene of *Schizothorax* species was sequenced and aligned with the 17 global sequences available in BOLD and Genbank database using the Clustal W program. This alignment allowed grouping of species into definite clusters. These *Schizothorax* species showed maximum sequence homology with the BOLD (99.3 to 99.9%) and NCBI (99.6 to 99.9%) reference sequences of those species. These four species have 14 haplotypes with haplotype diversity (*H*d) 0.9779 ± 0.027. The average value of nucleotide diversity (*P*i) was *P*i: 0.00878 ± 0.003.

The transitional substitutions are outnumbered the transversional substitutions. The estimated *T*s/*T*v bias (*R*) was =2.51. According to the Tajima *D* test and the Fu and Li D* and F* tests, the genetic variation between populations were not neutral (Tajima *D* = −0.957, *p* > .10; Fu and Li’s D* test statistic = 1.05942, *p* > .10; Fu and Li’s F* test statistic = 0.55331, *p* > .10). The negative values of Tajima’s D indicated that the genetic variations between populations were not neutral under the random effects of genetic drift and mutation which reflect the excess of external mutation.

## References

[CIT0001] Hajibabaei M, Smith MA, Janzen DH, Rodriguez JJ, Whitfield JB, Hebert PDN. 2006 A minimalist barcode can identify a specimen whose DNA is degraded. J Mol Ecol Notes. 6:959–964.

[CIT0002] Hebert PDN, Cywinska A, Ball SL, Waard JR. 2003 Biological identifications through DNA barcodes. Proc Biol Sci. 270:313–322. 12614582 10.1098/rspb.2002.2218 PMC1691236

[CIT0003] Jhingran VG. 1991 Fish and fisheries of India. India: Hindustan Publishing Corporation.

[CIT0004] Khan MF, Khattak MNK, He DK, Liang YY, Li C, Dawar F, Chen YF. 2015 Complete mitochondrial genome organization of *Schizothorax plagiostomus* (Teleostei: Cyprinidae) from Northern Pakistan. Mitochondrial DNA. 1–3. 26369352 10.3109/19401736.2015.1079829

[CIT0005] Kullander SO, Fang F, Delling B, Ahlander E. 1999 The fishes of the Kashmir Valley In: Nyman L, editor. River Jhelum, Kashmir Valley. Swedmar, Goteborg: Impact on the aquatic environment; p. 99–162.

[CIT0006] Mirza MR. 1991 A contribution to the systematics of the Schizothoracine fishes (Pisces: Cyprinidae) with the description of three new tribes. Pakistan J Zool. 23:339–341.

[CIT0007] Qi D, Guo S, Tang J, Zhao X, Liu J. 2007 Mitochondrial DNA phylogeny of two morphologically enigmatic fishes in the subfamily Schizothoracinae (Teleostei: Cyprinidae) in the Qinghai-Tibetan Plateau. J Fish Biol. 70:60–74.

[CIT0008] Sambrook J, Fritsch EF, Maniatis T. 1989 Molecular cloning: a laboratory manual, 2nd ed. New York: Cold Spring Harbor Laboratory, Cold Spring Harbor Laboratory Press; p. 1659.

[CIT0009] Tamura K, Stecher G, Peterson D, Filipski A, Kumar S. 2013 MEGA6: molecular evolutionary genetics analysis version 6.0. Mol Biol Evol. 30:2725–2729. 24132122 10.1093/molbev/mst197 PMC3840312

[CIT0010] Zhang DX, Hewitt GM. 1996 . Nuclear integrations: challenges for mitochondrial DNA markers. Trends Ecol Evol (Amst.). 11:247–251. 10.1016/0169-5347(96)10031-8 21237827

[CIT0011] Zhu YX, Chen Y, Cheng QQ, Qiao HY, Chen WM. 2012 The complete mitochondrial genome sequence of *Schizothorax macropogon* (Cypriniformes: Cyprinidae). Mitochondrial DNA. 24:237–239. 10.3109/19401736.2012.752478 23305330

